# New Arrangements

**Published:** 1885-12

**Authors:** E. W. Capron

**Affiliations:** Manager


### HALL'S JOURNAL OF HEALTH

Vol. 32. DECEMBER, 1885. No. 12.

### 1886!

### THIRTY-THIRD YEAR.

ESTABLISHED IN 1854.

A MONTHLY MAGAZINE,
TREATING OF HEALTH AND HOW TO KEEP IT.

*Household Affairs, with the latest important and best products in the line of*

**HEALTHY COOKING.**

*This department under the special charge of a Lady of experience.*

☞Under the management of no sect or pathy, we shall each month, from a careful examination of other publications, and from our own reports from a number of the best physicians of all pathies, cull that which we deem important and for the health and happiness of all.

TWELVE MORE PAGES OF READING MATTER WILL BE ADDED, making it th^ Largest and Cheapest Health Journal in the World.

**SUBSCRIPTION $1 00 PER YEAR.—10 CTS. PER COPY.**

HALL'S JOURNAL OF HEALTH will be clubbed with any other regular publication at 75 cts. per year. News Agents, Postmasters, Publishers and Booksellers are allowed 25 cts. on each yearly subscription sent us. Specimen copies sent on applicatien. Remittances should be made by postal note, check, or postage stamps.

**ADVERTISING RATES MADE KNOWN ON APPLICATION.**

JOURNAL OF HEALTH PUBLISHING CO.,

E. W. CAPRON, Manager. 75 & 77 BARCLAY ST., NEW YORK.

### NEW ARRANGEMENTS.

With the January number, the *Journal of Health* will be published under a new arrangement, and one in which we have made provisions for greatly extending its already large circulation and usefulness. It has long been known to many, having for these thirty-two full years made its appearance every month, with great regularity. It filled a place that was open for such a journal, and it has gained many friends, but it is such a journal as the family—every family in the country—needs and should have. It is our design now to make it a journal that no family can afford to be without. With the price still kept at ONE DOLLAR per year, we shall add ten or twelve pages of reading matter, and reading matter of the best quality for the home practice in every family.

With this view we have engaged several of the medical faculty, eminently practical in the profession, representing all the schools of practice, who will furnish us with the accounts of cases of interest for home treatment, also give an account of all that is going forward in the line of discovery in medical practice in all parts of the world, thus making it a *vade mecum* for both practitioner and family.

We shall also add what we have long wanted to give to the world, an important department to every number, the *Domestic Department*, which will pay particular attention to the cooking of food not only for the invalid but for the healthy living of all. While these departments will always be furnished, there will be others which our contributors will supply, to make this a first class health journal on which there can be no discount. In view of these extra outlays, we ask every subscriber to act as an agent—to at least send us one additional. That would double our already large circulation.

Of course, our space for advertising must be limited, but we know of no better mode than to say, first come first served. We shall be pleased to attend to those who call first.

JOURNAL OF HEALTH PUBLISHING CO.,
E. W. CAPRON, Manager.

